# The Diagnostic Challenge in Pulmonary Cryptococcosis Among Immunocompetent Patients: The ‘False-Negative’ Serum Cryptococcal Antigen

**DOI:** 10.3390/jof12080609

**Published:** 2026-08-14

**Authors:** Miaojuan Zhu, Nishan Deng, Hanxiang Nie

**Affiliations:** Department of Respiratory and Critical Care Medicine, Renmin Hospital of Wuhan University, 99 Zhangzhidong Road, Wuchang District, Wuhan 430060, China; zhumiaoj@whu.edu.cn (M.Z.); nishandeng@whu.edu.cn (N.D.)

**Keywords:** pulmonary cryptococcosis, immunocompetent, serum cryptococcal antigen, false-negative

## Abstract

Objective: To delineate the demographic, clinical, and radiological features of immunocompetent patients with pulmonary cryptococcosis (PC) who test negative for serum cryptococcal antigen (CrAg), a subgroup that poses a diagnostic challenge. Methods: Participants were stratified into CrAg-positive (*n* = 128) and CrAg-negative (*n* = 71) cohorts. Comparative analyses were performed on clinical presentation, chest computed tomography (CT) imaging features, and laboratory parameters. Results: Patients with false-negative CrAg results were more likely to be asymptomatic (46.5% vs. 20.3% in the CrAg-positive group; *p* < 0.05). Their chest CT scans typically revealed solitary pulmonary nodules, frequently unilateral and located in the right lung, with a notable absence of pleural effusion or lymphadenopathy. Laboratory findings in the CrAg-negative group were characterized by a lower neutrophil ratio and erythrocyte sedimentation rate. Furthermore, higher CrAg titers were significantly correlated with the presence of respiratory symptoms, specific CT signs (e.g., air bronchogram, lymphadenopathy), and elevated systemic inflammatory markers. Conclusions: False-negative serum CrAg in immunocompetent PC patients is characterized by an asymptomatic presentation and a solitary nodule on imaging, with normal laboratory parameters. CrAg titers may reflect disease severity and warrant prospective evaluation as a potential biomarker for guiding antifungal therapy.

## 1. Introduction

Cryptococcus, especially the basidiomycetous yeasts of the Cryptococcus neoformans or Cryptococcus gattii species complexes, is the main pathogen causing global cryptococcosis [1]. Cryptococcosis is an invasive fungal disease. Since Cryptococcus is usually obtained by inhalation, the lung is the portal of entry [2]. Few cases of infection are obtained through solid organ transplantation [3]. After inhaling Cryptococcus neoformans, meningitis caused by pulmonary bloodstream infection mostly occurs in AIDS patients, causing up to 180,000 deaths worldwide each year, exceeding tuberculosis and malaria in annual deaths [4,5].

More than half of patients with pulmonary cryptococcosis are without HIV or immune diseases. In addition, about 60% of non-HIV patients with pulmonary cryptococcosis have no previous underlying disease [6]. Pulmonary cryptococcosis in HIV-negative patients is most common in China (84% to 96%) compared to other regions, with about 70% of patients without any previous disease [7,8,9].

The clinical manifestations of pulmonary cryptococcosis are often nonspecific and may include cough, fever, dyspnea, chest pain, weight loss, and headache. Imaging findings vary, with lesions most frequently appearing as single or multiple nodules and patchy opacities. The gold standard for diagnosis is microbial culture from infected tissue. However, cultures may yield negative results in approximately 40% of immunocompetent patients with histopathologically confirmed solitary pulmonary lesions. Cryptococcal antigen detection (CrAg), based on antibody–antigen interactions, employs latex agglutination or lateral flow assays (LFA) of serum or cerebrospinal fluid. CrAg testing is a key tool for clinical screening of cryptococcal infection, and a positive serum cryptococcal capsular antigen test strongly supports the diagnosis [10,11].

The sensitivity of serum or cerebrospinal fluid cryptococcal capsular antigen (CrAg) testing in cryptococcal meningitis can reach as high as 99% [4,5]. However, it has been reported that pulmonary cryptococcosis accounts for approximately 69% of all cryptococcosis cases, and only about 64% of these patients exhibit normal immune function [9]. In summary, among immunocompetent patients with pulmonary cryptococcosis, false-negative serum CrAg occurs in approximately one-third of cases, thereby reducing diagnostic accuracy and potentially affecting treatment choices. Our study sought to delineate the demographic, clinical, and radiological characteristics of this patient subgroup, with the goal of providing diagnostic references for clinicians.

## 2. Materials and Methods

### 2.1. Patients

The clinical data of all patients with pulmonary cryptococcosis admitted to the Department of Respiratory and Critical Care Medicine of Renmin Hospital of Wuhan University from January 2017 to November 2025 were collected. Inclusion criteria: (1) age > 18 years; (2) positive for Cryptococcus by special staining of lung histopathology (ink staining/hexamine silver method), pathogenic smear, or fungal culture of sterile lung tissue specimens; (3) Cryptococcus was detected by sputum, bronchoalveolar lavage fluid smear, culture, or high-throughput nucleic acid sequencing. (4) Have complete medical records. Exclusion criteria: (1) human immunodeficiency virus infection, solid organ transplantation, bone marrow transplantation, long-term use of glucocorticoids or immunosuppressive agents, autoimmune diseases, malignant tumors receiving chemotherapy in the past 2 years, chronic kidney disease stage 4 or 5, decompensated cirrhosis; (2) combined with cryptococcal meningitis or other extrapulmonary cryptococcal infection; (3) Incomplete medical records.

### 2.2. Data and Information Collection

We collected patient information indiscriminately, including (1) demographic characteristics: gender, age, history of suspected exposure, and history of smoking and drinking; (2) Clinical features: asymptomatic, fever, cough and expectoration, chest pain, dyspnea/chest tightness, hemoptysis, fatigue, weight loss, lung moist rales, dry rales, reduced breath sounds and coarse breath sounds; (3) Past medical history: diabetes, coronary heart disease, thyroid disease and cirrhosis/hepatitis; (4) Imaging features: pulmonary lobe distribution, relationship with pleura, concurrent imaging signs and lesion characteristics; (5) Laboratory examination: blood routine, erythrocyte sedimentation rate, C-reactive protein(CRP), procalcitonin(PCT); (6) Serum cryptococcal antigen (CrAg): Serum CrAg testing was performed as follows. The serum cryptococcal antigen was detected using a lateral flow immunochromatographic assay (IMMY, Norman, OK, USA), which is based on a sandwich immunoassay targeting the capsular polysaccharide glucuronoxylomannan (GXM). The analytical sensitivity of this assay is approximately 0.5–1.0 ng/mL according to the manufacturer’s instructions. Positive samples were further semi-quantified by serial twofold titrations to determine the CrAg titer. All patients were tested using this lateral flow immunochromatographic assay exclusively; latex agglutination was not employed in this study, eliminating the concern of lower analytical sensitivity associated with the latex agglutination method. (7) For asymptomatic patients, indications for chest imaging included routine health check-ups (accounting for approximately 60% of asymptomatic cases) and incidental findings during hospitalizations for non-pulmonary conditions.

### 2.3. Statistical Analysis

Data were analyzed using SPSS 24.0 (IBM Corporation, Armonk, NY, USA) and R statistical software (R Foundation for Statistical Computing, Vienna, Austria). Continuous variables were tested for normality (Shapiro–Wilk test) and homogeneity of variance (Levene’s test). Normally distributed data were expressed as mean ± standard deviation ($\bar{x}$ $\bar{x}$ ± SD) and compared using the independent samples *t*-test. Non-normally distributed data were reported as median (interquartile range) [M (P25, P75)] and analyzed with the Wilcoxon rank-sum test. Categorical data were presented as frequency (percentage) [*n* (%)] and evaluated by the chi-square test. A two-tailed *p*-value < 0.05 was considered statistically significant.

## 3. Result

### 3.1. Clinical Manifestation of Pulmonary Cryptococcosis in Immunocompetent Patients

Among the 63 asymptomatic patients, 38 (60.3%) were identified through routine health screening, while the remaining 25 were incidentally detected during evaluations for unrelated medical conditions. CrAg-negative patients were significantly more likely to be asymptomatic and had a lower prevalence of cough/sputum production and chest pain compared to CrAg-positive patients (*p* < 0.05 for all). They also had a lower frequency of diabetes mellitus (*p* < 0.05). No significant differences were found between the groups regarding age, sex, tobacco use, alcohol consumption, or pigeon exposure (Table 1).

### 3.2. CT Features of Pulmonary Cryptococcosis in Immunocompetent Patients

Compared with CrAg-positive patients (*p* < 0.05), lung CT lesions in CrAg-negative patients mostly occurred in the unilateral lungs, mainly in the right lung. The nature of the lesion is mostly manifested as a single consolidation nodule in the right lung away from the pleura, and there are almost no accompanying signs such as pleural effusion, cavity, and mediastinal lymph node enlargement (Table 1).

### 3.3. Laboratory Findings of Pulmonary Cryptococcosis in Immunocompetent Patients

Compared with CrAg-positive patients, those who were CrAg-negative had a significantly lower proportion of neutrophils and a lower erythrocyte sedimentation rate (ESR), but a higher proportion of lymphocytes; however, all values remained within normal limits. No significant differences were observed in white blood cell (WBC) count, C-reactive protein (CRP), or procalcitonin (PCT) levels, all of which were also within the normal range (Table 2).

### 3.4. Influencing Factors of Serum CrAg Titers

The median CrAg titers for semiquantitative score categories (1+ to 3+) were 1:10 (interquartile range [IQR], 1:5 to 1:20) in the CrAgSQ 1+ category. 1:40 (IQR, 1:20 to 1:80) in the CrAgSQ 2+ category, and 1:640 (IQR, 1:160 to 1:2560) in the CrAgSQ 3+ category. Elevated Cough and sputum, Bronchial inflation sign, Lymphadenopathy, ground glass/solid shadow, WBC (×10^9^), Neutrophil count (×10^9^), and Neutrophil ratio (%) were more common in the CrAgSQ 3+ category. Pleural traction signs, burr signs, and elevated erythrocyte sedimentation rate were more common in the CrAgSQ 2+ category. The increase in lymphocyte ratio (%) was more common in the CrAgSQ 2+ category (*p* < 0.05) (Table 3).

### 3.5. Diagnostic Efficacy of Serum Cryptococcal Capsular Antigen

**Figure 1:**
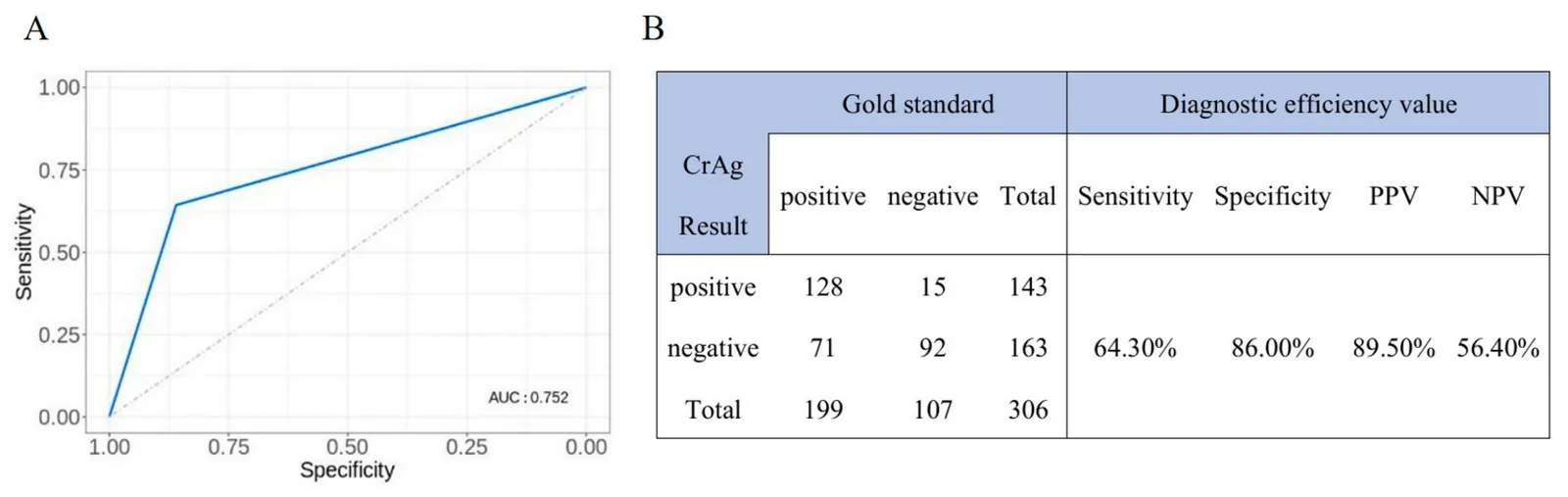
ROC curve analysis of serum CrAg assay. The solid blue line represents the ROC curve of the serum CrAg assay, with an area under the curve (AUC) of 0.752(95% CI: 0.695–0.808). (**A**) The dashed diagonal gray line is the reference line (AUC = 0.5), indicating a test with no discriminatory ability. (**B**) CrAg diagnostic index value.

To assess the diagnostic performance of serum CrAg, we evaluated 199 patients with pulmonary cryptococcosis and 107 with non-cryptococcal diseases (negative controls). The negative control group comprised 107 patients with non-cryptococcal pulmonary diseases, including bacterial pneumonia (*n* = 32), pulmonary tuberculosis (*n* = 28), malignant lung tumors (*n* = 25), and benign pulmonary nodules (*n* = 22). All controls underwent chest CT imaging, and their radiological findings (solitary or multiple nodules/masses, ground-glass opacities, etc.) were comparable to those of the cryptococcosis cases. The “Gold Standard” in Figure 1B refers to the definitive diagnostic criteria used for case classification: for the cryptococcosis group, diagnosis was confirmed by histopathology (special stains), fungal culture from sterile sites, or high-throughput nucleic acid sequencing; for the control group, diagnoses were confirmed by bacterial culture, Mycobacterium tuberculosis culture/PCR, histopathology (for malignancies), or long-term clinical and radiological follow-up (for benign nodules). The test was positive in 128 (64.3%) of the cryptococcosis patients and in 15 of the non-cryptococcal patients. This yielded a sensitivity of 64.3%, a specificity of 86.0%, a positive predictive value (PPV) of 89.5%, and a negative predictive value (NPV) of 56.40%. The area under the receiver operating characteristic curve (AUC) was 0.752 (95% CI: 0.695–0.808) (Figure 1).

## 4. Discussion

Cryptococcosis remains a significant global health concern, contributing to considerable morbidity and mortality worldwide [12,13]. Although traditionally considered an opportunistic infection predominantly affecting immunocompromised individuals—such as AIDS patients, those with malignancies, or recipients of immunosuppressive therapy—there is growing evidence of its increasing incidence among immunocompetent hosts. In non-HIV populations, pulmonary cryptococcosis (PC) is the most common clinical form of cryptococcal infection [14].

Diagnosing PC poses considerable challenges due to its nonspecific clinical and radiological features [15,16], often resulting in delayed or incorrect diagnoses [17]. These diagnostic difficulties highlight the need for a better characterization of PC in immunocompetent populations.

To address this gap, we analyzed 199 immunocompetent patients with confirmed pulmonary cryptococcosis. This cohort represents one of the largest samples of immunocompetent PC patients in China to date, substantially exceeding the sample sizes reported in previous studies by Xinqiang et al. [18] and Xie et al. [19], thereby reducing potential statistical biases. Moreover, our study applied more stringent inclusion criteria than earlier investigations by Lin et al. [20] and Hou et al. [21], systematically excluding not only HIV-positive individuals but also patients with autoimmune diseases or malignancies that could impair immune function. This rigorous approach strengthens the validity of our findings regarding PC in truly immunocompetent hosts.

Our results showed that immunocompetent PC patients had a mean age of 48.97 ± 13.19 years, with a notable male predominance (male-to-female ratio = 126:73), consistent with earlier reports by Lin et al. [20]. Female patients were significantly older at presentation than males (53.05 ± 11.51 vs. 43.61 ± 13.56 years; *p* < 0.001). The most common symptom was cough and expectoration (n = 96, 48.24%), while a considerable proportion of patients were asymptomatic (n = 63, 31.66%) [22,23]. Previous studies have also indicated that immunocompetent individuals with PC are more likely to be asymptomatic compared to immunocompromised patients [24,25]. Hemoptysis was the least frequent symptom (n = 6, 3.02%), which may help differentiate PC from conditions such as tuberculosis, bronchiectasis, or lung tumors [26]. Notably, physical examination rarely revealed dry rales, a hallmark of bronchial asthma. Fever was also uncommon (n = 28, 14.07%). Studies suggest that fever is more frequent in extrapulmonary cryptococcosis (68.9%) than in pulmonary cases (15.2%) [27] and is associated with the patient’s immune status, being more common in immunocompromised hosts [28]. Additionally, most immunocompetent PC patients had no significant comorbidities, suggesting that underlying conditions play a limited role in the pathogenesis of PC in this population.

High-resolution computed tomography (HRCT) is essential for evaluating pulmonary diseases. Previous studies have reported that the most common radiological manifestation of PC is multiple nodules or masses [29]. For instance, Xinqiang et al. [18] observed nodules or masses in 55.2% of cases, consolidation in 12.1%, and mixed patterns in 32.8%, with lesions predominantly located in the lower lobes (69.0%) and peripheral regions (94.8%). Mediastinal lymphadenopathy and pleural effusion were uncommon. Similarly, Su et al. [30] reported that PC frequently involved the right lower lobe. Our findings align with these reports: 58.79% of patients had bilateral involvement, with a lower lobe predominance (n = 110, 55.28%). Nodules or masses were observed in 112 patients (56.28%), while cavitation (n = 20, 10.05%), pleural effusion (n = 8, 4.02%), and lymphadenopathy (n = 9, 4.52%) were rare. These imaging features—such as bilateral lower lobe distribution (particularly right-sided), multiple nodules, or masses—should prompt consideration of PC in the differential diagnosis.

Among the immunocompetent PC patients, 128 were CrAg-positive, and 71 were CrAg-negative. Symptoms such as cough and expectoration (n = 71, 55.47%), chest pain (n = 26, 20.31%), and comorbidities like diabetes (n = 15, 11.72%) were more frequent in the CrAg-positive group. In contrast, most CrAg-negative patients were asymptomatic (n = 33, 46.48%) and had fewer complications. These findings suggest that asymptomatic immunocompetent patients with suspected PC are more likely to have a negative CrAg result, underscoring the need for clinical vigilance to avoid missed diagnoses. Previous studies have identified type 2 diabetes as a risk factor and prognostic indicator in HIV-negative, non-transplant cryptococcosis cases [31]. The influence of diabetes on the pathogenesis, outcomes, and CrAg expression in PC warrants further investigation.

Radiologically, the CrAg-positive group more frequently had multiple lesions (n = 84, 65.63%), bilateral distribution (n = 51, 39.84%), cavitation (n = 17, 13.28%), pleural effusion (n = 8, 6.25%), lymphadenopathy (n = 9, 7.03%), and ground-glass opacities or consolidation (n = 57, 44.53%). In contrast, CrAg-negative patients tended to have solitary lesions (n = 38, 53.52%), often in the right lung (n = 39, 54.93%), with no lymphadenopathy or pleural effusion. Lesions in this group were primarily nodules or masses (n = 35, 49.30%). These patterns indicate that CrAg-positive patients often present with more diffuse and complicated disease, whereas CrAg-negative cases exhibit more localized imaging findings.

Several interrelated factors may account for the higher frequency of false-negative serum CrAg results observed in immunocompetent patients with pulmonary cryptococcosis. First, the fungal burden in these patients is typically lower than in immunocompromised hosts, as the infection often manifests as a solitary pulmonary nodule with limited local extension. This localized confinement reduces the release of capsular polysaccharide antigens into the bloodstream, making them less detectable by serum assays [23]. Second, the intact cellular immune response in immunocompetent individuals effectively contains the pathogen within the lungs, minimizing hematogenous dissemination and thereby limiting systemic antigenemia. This is consistent with our radiological observation that CrAg-negative patients rarely presented with pleural effusion or lymphadenopathy—features that typically reflect more extensive disease. Third, strain-specific differences in capsular polysaccharide production and shedding may influence antigen detectability; Cryptococcus neoformans and Cryptococcus gattii species complexes exhibit considerable variability in GXM expression, which could affect assay performance [32]. Finally, inherent limitations of laboratory methodologies must be considered. While the lateral flow immunoassay used in this study has high analytical sensitivity (0.5–1.0 ng/mL), false-negative results can still occur due to the prozone (hook) effect in undiluted samples with extremely high antigen concentrations [33], as well as variations in specimen storage and handling [34]. Collectively, these pathophysiological and methodological factors underscore the complexity of interpreting a negative serum CrAg result in immunocompetent patients and reinforce the importance of integrating clinical, radiological, and microbiological findings for accurate diagnosis.

Laboratory findings revealed a higher neutrophil ratio (64.55 ± 11.22%) and erythrocyte sedimentation rate (31.0 ± 31.32 mm/h) in the CrAg-positive group, while the CrAg-negative group had a higher lymphocyte ratio (30.26 ± 8.56% vs. 25.22 ± 9.98%). Given that lymphocytes reflect host immune status [35], the more limited disease and asymptomatic presentation in CrAg-negative patients may be associated with better-preserved cellular immunity.

In summary, for a patient presenting with a solitary pulmonary nodule in the absence of clinical symptoms and with normal laboratory findings, and after exclusion of conditions such as malignancy and tuberculosis that may exhibit similar imaging features, pulmonary cryptococcosis may still be considered a potential diagnosis despite a negative CrAg result.

To further explore factors influencing CrAg titers, patients were stratified by semiquantitative CrAg (CrAgSQ) scores. We found that symptoms (cough and sputum), imaging features (bronchial inflation sign, lymphadenopathy, ground-glass/consolidation), and laboratory parameters (elevated WBC, neutrophil count, and neutrophil ratio) were positively correlated with higher antibody titers. Previous studies have suggested that the size and number of CT lesions may relate to treatment response and CrAg levels [36]. Future prospective studies should evaluate how changes in symptoms, imaging findings, and laboratory markers correlate with CrAg titers during antifungal therapy in immunocompetent patients.

Serum CrAg testing is a key diagnostic tool for PC. In our immunocompetent cohort, it demonstrated a sensitivity of 64.3%, specificity of 86.0%, positive predictive value of 89.5%, and negative predictive value of 56.4%. The area under the receiver operating characteristic curve was 0.752 (95% CI: 0.695–0.808). These results are largely consistent with previous reports; for example, Yan et al. [37] reported a sensitivity of 68.8% and specificity of 97.7%, while another study found a sensitivity of 93.3% [38]. Based on our CrAg subgroup analyses, we speculate that factors such as immune status and imaging characteristics may influence CrAg sensitivity in immunocompetent patients. Therefore, diagnosis should not rely solely on CrAg testing to avoid false negatives. That said, its high specificity is valuable for ruling out non-cryptococcal disease and reducing unnecessary additional investigations.

Recently, lateral flow immunoassays (LFA) targeting the major capsular polysaccharide antigen glucuronoxylomannan (GXM) have emerged as a complementary diagnostic tool. This method is simple, rapid, equipment-independent, and particularly suitable for resource-limited settings [39]. Chen et al. [40] evaluated CrAg-LFA in bronchoalveolar lavage fluid (BALF) from HIV-negative PC patients and reported a BALF sensitivity of 88.1% and specificity of 99.6%, while serum CrAg-LFA showed 90.5% sensitivity and 99.6% specificity. Notably, combining BALF and serum LFA achieved 100% sensitivity and negative predictive value, with a specificity of 99.1% [40]. These findings suggest that LFA, especially when combined with BALF testing, could effectively complement serum CrAg in patients with localized lesions or negative serum results. Nonetheless, serum CrAg testing remains valuable for excluding non-cryptococcal disease due to its high specificity (86.0% in our study).

All 199 patients with confirmed pulmonary cryptococcosis received antifungal therapy. Treatment regimens were individualized based on disease severity and lesion extent, with fluconazole monotherapy (400 mg daily) being the most commonly prescribed regimen, while patients with diffuse or more severe disease received a combination of amphotericin B and fluconazole. Treatment duration ranged from 3 to 12 months depending on clinical response. We monitored the clinical outcomes in a cohort of patients. In two cases with an initial CrAg titer of 1:640, a gradual decline in titer was observed following the initiation of antifungal therapy, accompanied by radiographic evidence of lesion resolution on lung CT. However, disease recurrence occurred three months after treatment completion, with the CrAg titer rising again to 1:640. Repeat imaging demonstrated an increased extent of pulmonary infiltration. In contrast, no relapses were observed among CrAg-negative patients following antifungal treatment. Although these observations suggest a possible association between CrAg titers and disease activity, our retrospective data are insufficient to establish definitive therapeutic recommendations. CrAg titers should be regarded as a promising candidate biomarker that merits validation in prospective studies designed to assess its utility in guiding antifungal therapy.

This study has several limitations. First, its single-center design may limit the generalizability of findings to populations of different ethnic or geographic backgrounds. Second, the retrospective nature introduces the possibility of data collection bias. Third, future multicenter prospective studies are needed to validate and extend our findings. Fourth, the study period overlapped with the COVID-19 pandemic (2020–2023); however, only a minority of cases were diagnosed during this period, and we were unable to reliably assess potential associations between SARS-CoV-2 co-infection and pulmonary cryptococcosis due to the retrospective design and limited case numbers. This question warrants further investigation in future prospective cohorts. Fifth, although histopathological patterns of cryptococcal infection (ranging from abundant yeast forms to well-formed granulomas with sparse intracellular organisms) may theoretically influence antigen release, the retrospective nature of this study and the heterogeneity of biopsy specimens (including surgical resections, percutaneous lung biopsies, and bronchoscopic biopsies) prevented a systematic, quantitative correlation analysis between pathological patterns and serum CrAg results. This represents an important avenue for future prospective histopathological studies. Sixth, molecular speciation was not performed for all culture-positive isolates, precluding analysis of potential differences in CrAg sensitivity between Cryptococcus neoformans and Cryptococcus gattii species complexes. This is a notable limitation, as strain-specific variability in capsular polysaccharide expression has been previously described. Seventh, comprehensive long-term follow-up data were not uniformly available for all patients due to the retrospective study design. While we observed an association between CrAg titer decline and radiographic improvement in a subset of patients (approximately 30% of the cohort), the lack of systematic follow-up for all cases limits our ability to draw definitive conclusions regarding long-term treatment outcomes. Prospective studies with standardized follow-up protocols are needed to validate these observations.

## 5. Conclusions

Pulmonary cryptococcosis in immunocompetent, CrAg-negative individuals is characterized by an asymptomatic presentation and a solitary nodule on imaging, with non-specific laboratory parameters. Our observations suggest that CrAg titers may correlate with disease severity and treatment response, indicating their potential role as a prognostic biomarker that warrants further prospective evaluation for guiding therapeutic decisions.

## Figures and Tables

**Table 1:** Clinical characteristics of pulmonary cryptococcosis in immunocompetent patients [n, (%)].

Clinical Characteristics	CrAg Results			
	Positive (n = 128)	Negative (n = 71)	χ^2^/|t|/|Z|	*p*-Value
Demographic characteristics				
Age (Mean ± SD ^†^)	48.62 ± 13.58	49.62 ± 12.50	0.513	0.609
Male	84 (65.63%)	42 (59.15%)	0.823	0.364
Female	44 (34.38%)	29 (40.85%)		
Tobacco use	22 (17.19%)	10 (14.08%)		
Alcohol consumption	24 (18.75%)	11 (15.49%)	0.334	0.563
Pigeon exposure history	4 (3.13%)	0 (0.00%)	-	0.299
Clinical characteristics				
Cough with sputum production	71 (55.47%)	25 (35.21%)	7.506	0.006
Asymptomatic	30 (23.44%)	33 (46.48%)	11.206	0.001
Pyrexia	22 (17.19%)	6 (8.45%)	2.883	0.090
Chest pain	26 (20.31%)	3 (4.23%)	-	0.002
Dyspnea/chest tightness	25 (19.53%)	10 (14.08%)	0.935	0.334
Hemoptysis	3 (2.34%)	3 (4.23%)	-	0.457
Fatigue	20 (15.63%)	12 (16.90%)	0.055	0.814
Weight loss	13 (10.16%)	3 (4.23%)	2.173	0.140
Crackles	11 (8.59%)	6 (8.45%)	0.001	0.972
Rhonchi	0 (0.00%)	0 (0.00%)	-	-
Decreased breath sounds	13 (10.16%)	8 (11.27%)	0.060	0.807
Coarse breath sounds	39 (30.47%)	17 (23.94%)	0.962	0.367
Past medical history				
Diabetes mellitus	15 (11.72%)	2 (2.82%)	-	0.031
Coronary artery disease, CAD	6 (4.69%)	2 (2.82%)	0.414	0.520
Thyroid disorders	3 (2.34%)	3 (4.23%)	0.523	0.470
Pulmonary CT features				
Pulmonary lobe distribution				
Left lung	28 (21.88%)	16 (22.54%)	0.043	0.836
Right lung	48 (37.50%)	39 (54.93%)	6.570	0.010
Both lungs	51 (39.84%)	15 (21.13%)	7.218	0.007
Upper lobe	14 (10.94%)	14 (19.72%)	2.912	0.088
Middle lobe	2 (1.56%)	1 (1.41%)	0.007	0.932
Lower lobe	72 (56.25%)	38 (53.52%)	0.138	0.711
Multilobar involvement	21 (16.41%)	10 (14.08%)	0.925	0.336
Relationship with pleura				
Pleural adjacency	46 (35.94%)	23 (32.39%)	0.253	0.615
Distant from pleura	73 (57.03%)	43 (60.56%)	0.234	0.628
Pleural indentation	16 (12.50%)	8 (11.27%)	0.065	0.798
Concurrent signs				
Halo sign	34 (26.56%)	12 (16.90%)	2.398	0.121
Spiculation	35 (27.34%)	21 (29.58%)	0.113	0.737
Tree-in-bud sign	34 (26.56%)	18 (25.35%)	0.035	0.852
Cavitation	17 (13.28%)	3 (4.23%)	4.143	0.042
Air bronchogram	28 (21.88%)	11 (15.49%)	1.180	0.277
Pleural effusion	8 (6.25%)	0 (0.00%)	4.623	0.032
Lymphadenopathy	9 (7.03%)	0 (0.00%)	5.229	0.022
Characteristics of lesions				
Solitary lesion	44 (34.38%)	38 (53.52%)	6.910	0.009
Multiple lesions	84 (65.63%)	33 (46.48%)		
Nodules/masses	57 (44.53%)	55 (77.46%)	20.131	<0.001
Solitary nodule/mass	22 (17.19%)	35 (49.30%)	7.076	0.008
Multiple nodules/masses	34 (26.56%)	19 (26.76%)		
Ground-Glass Opacities/Consolidations	57 (44.53%)	12 (16.90%)	15.392	<0.001
Focal GGO/consolidation *	22 (17.19%)	3 (4.23%)	1.218	0.270
Multifocal GGO/consolidations *	33 (25.78%)	9 (12.68%)		
Mixed GGO and solid components *	16 (12.50%)	6 (8.45%)	0.802	0.370

^†^ Standard deviation, SD. * The categories under “Ground-Glass Opacities/Consolidations” (i.e., “Focal GGO/consolidation,” “Multifocal GGO/consolidations,” and “Mixed GGO and solid components”) are mutually exclusive classifications based on the predominant radiological pattern of each patient’s pulmonary lesions.

**Table 2:** Laboratory Parameters in Pulmonary Cryptococcosis [Mean ± SD].

Laboratory Results	CrAg (+)	CrAg (−)	t/z/x^2^	*p*-Value	Normal Range
WBC ^†^ (×10^9^)	7.13 ± 2.38	6.41 ± 2.27	1.378	0.372	4–10
Neutrophil count (×10^9^)	4.76 ± 2.32	3.89 ± 2.17	1.722	0.789	1.2–6.8
Lymphocyte count (×10^9^)	1.71 ± 0.69	1.86 ± 0.56	1.028	0.307	0.8–4.0
Monocyte count (×10^9^)	0.57 ± 0.27	0.50 ± 0.17	1.503	0.137	0.3–0.8
Eosinophilic count (×10^9^)	0.15 ± 0.20	0.14 ± 0.17	0.358	0.721	0.02–0.5
Basophil count (×10^9^)	0.04 ± 0.04	0.03 ± 0.02	0.807	0.422	0–0.1
Neutrophil percentage (%)	64.55 ± 11.22	58.79 ± 9.68	2.424	0.017	43–76
Lymphocyte percentage (%)	25.22 ± 9.98	30.26 ± 8.56	2.389	0.019	17–48
Monocyte percentage (%)	9.21 ± 9.18	7.81 ± 1.27	0.841	0.402	4–10
Eosinophil percentage (%)	1.97 ± 1.55	2.41 ± 2.00	1.275	0.206	0–1
basophil percentage (%)	0.51 ± 0.52	0.51 ± 0.33	0.016	0.988	0.5–5
CRP ^‡^ (mg/L)	17.39 ± 40.53	12.22 ± 27.38	0.497	0.621	<10
ESR ^§^ (mm/h)	31.04 ± 31.32	7.63 ± 4.90	3.733	<0.001	Male 0–15
					Female 0–20
PCT ^¶^ (ng/mL)	0.06 ± 0.01	0.10 ± 0.19	1.187	0.240	<0.15

^†^ White blood cell, WBC; ^‡^ C reactive protein, CRP; ^§^ Erythrocyte sedimentation rate, ESR; ^¶^ Procalcitonin, PCT.

**Table 3:** Influencing factors of CrAg titer [n, (%)], [Mean ± SD].

Influencing Factors	CrAg Titer (CrAgSQ)			
	1+ (n = 24)	2+ (n = 14)	3+ (n = 17)	*p*-Value
Cough with sputum production	9 (37.50%)	10 (71.43%)	15 (88.24%)	0.002
Pleural indentation sign	3 (12.50%)	7 (50.00%)	5 (29.41%)	0.041
Spiculated margin	11 (45.83%)	12 (85.71%)	12 (70.59%)	0.031
Air bronchogram sign	4 (16.67%)	9 (64.29%)	14 (82.35%)	<0.001
Lymphadenopathy	0 (0.00%)	4 (28.57%)	5 (29.41%)	0.003
Ground-glass opacity/Consolidation	6 (25.00%)	6 (42.86%)	11 (64.71%)	0.0376
WBC ^†^ (×10^9^)	6.03 ± 1.05	7.69 ± 3.02	8.23 ± 2.59	0.004
Neutrophil count (×10^9^)	3.57 ± 1.02	5.41 ± 2.99	5.91 ± 2.35	0.001
Neutrophil percentage (%)	58.58 ± 10.17	67.60 ± 10.61	70.47 ± 9.29	0.001
Lymphocyte percentage (%)	30.57 ± 8.89	22.40 ± 9.80	19.98 ± 8.12	0.001
ESR ^‡^ (mm/h)	19.42 ± 26.50	42.17 ± 36.29	39.11 ± 32.12	0.044

^†^ White blood cell, WBC; ^‡^ Erythrocyte sedimentation rate, ESR.

## Data Availability

The original data for this article are available from the author.
